# Inversion of kiwifruit canopy nitrogen using UAV multispectral technology and ensemble learning

**DOI:** 10.3389/fpls.2026.1785943

**Published:** 2026-03-11

**Authors:** Bing Zhou, Yunshuang Wang, Jinheng Zhang, Xiaoyi Bai, Mingkui Tian, Ruinan Guo

**Affiliations:** 1 College of Science, Yunnan Agricultural University, Kunming, China; 2 College of Big Data, Yunnan Agricultural University, Kunming, China; 3 Modern Education Center, Yunnan Agricultural University, Kunming, China

**Keywords:** ensemble learning, kiwifruit, mountainous orchard, nitrogen inversion, SHAP, UAV multispectral technology

## Abstract

Accurate nitrogen monitoring is a key prerequisite for the high-quality, high-yield, and sustainable cultivation of mountainous kiwifruit, yet the complex topography of mountainous regions and the unique vine canopy structure of kiwifruit limit the applicability of traditional monitoring methods. In this study, a low-latitude, high-altitude mountainous kiwifruit orchard in Yunnan, China, was selected as the study area, with a focus on the fruit expansion stage (August). We integrated UAV multispectral technology and ensemble learning algorithms to perform canopy nitrogen inversion. Canopy images were acquired via UAV, and 278 experimental plots were synchronized with ground-based measured nitrogen data collection. Twenty-five spectral variables, five single models, and three ensemble learning models were constructed, and the SHAP method was employed to analyze the contribution of each spectral feature to nitrogen inversion. The results showed that eleven spectral variables were significantly correlated with nitrogen content, PLSR was the best single model, and the Boosting ensemble model had the best inversion accuracy (R2 = 0.89, RMSE = 0.50, RPD = 2.99). The heatmap generated by the model clearly depicts the spatial distribution of nitrogen. This study confirms the feasibility of coupling UAV multispectral technology with ensemble learning algorithms for nitrogen inversion, optimizes the nitrogen inversion technical system for mountainous vine fruits, and provides theoretical and technical support for the popularization of smart agriculture in mountainous orchards.

## 1 Introduction

Kiwifruit (Actinidia chinensis Planch.) is rich in high-value nutrients, including vitamins, dietary fiber, and antioxidants (Änäkkälä et al., 2022; Fan et al., 2024). As the origin, research center, and largest producer of kiwifruit globally, China has several advantageous cultivation regions. Yunnan Province, leveraging its unique low-latitude and high-altitude geographic and climatic advantages, has developed a concentrated high-quality mountainous kiwifruit production area (Faostat, 2020; Fu et al., 2021). The abundant light and heat resources in the region have laid a good foundation for fruit quality enhancement, but the complex topographic conditions of mountainous orchards also bring many challenges for field precision management. Nitrogen, a “life element” for plant growth and development, directly regulates key physiological processes of kiwifruit, such as photosynthetic efficiency, cell division, and photosynthate accumulation. It is crucial for fruit yield formation, sugar accumulation, and flavor quality development (Giletto et al., 2025). Insufficient nitrogen supply will lead to plant yellowing, growth retardation, and small fruits, while excessive nitrogen supply will trigger uncontrolled vegetative growth, reduced disease resistance, and aggravated agricultural non-point source pollution (Huang et al., 2023). Therefore, accurate and non-destructive monitoring of canopy nitrogen is a core prerequisite for the high-quality, high-yield, and sustainable cultivation of mountainous kiwifruit.

Traditional nitrogen monitoring methods mainly include manual visual estimation and laboratory chemical analysis. Manual visual estimation relies on growers’ empirical judgment, which is subjective and lagging, often leading to missed optimal nutrient regulation timings (Ihuoma and Madramootoo, 2020). Laboratory chemical analysis requires destructive plant sampling, with cumbersome and time-consuming operations, making it difficult to meet the demand for real-time dynamic nitrogen monitoring in orchards (Jia et al., 2024; Jiang et al., 2024). Although handheld plant nutrient meters enable non-destructive on-site measurement, their sparse sampling points and limited spatial coverage result in inadequate spatial representativeness, failing to accurately reflect nitrogen spatial heterogeneity in large-scale orchards. With the rapid advancement of precision agriculture, Unmanned Aerial Vehicle (UAV) remote sensing technology equipped with multispectral sensors has emerged as a core tool for inverting crop physiological and biochemical parameters, thanks to its high spatiotemporal resolution, non-destructiveness, and operational flexibility (Jiao et al., 2021). This technology can rapidly and extensively acquire canopy spectral reflectance information, providing rich and continuous data for nitrogen content inversion. It effectively overcomes the limitations of traditional methods in spatiotemporal coverage and monitoring accuracy, enabling efficient and large-scale nitrogen monitoring in orchards (Kang et al., 2021).

At present, UAV-based multispectral crop nitrogen inversion has formed one of the important technical paths of “multi-feature fusion and machine learning modeling” (Kokkora et al., 2023). As the basic spectral information, single-band reflectance directly characterizes the absorption and reflection properties of the canopy to different wavelengths of light, which is the core basic feature of nitrogen inversion (Lafond, 2023); Vegetation indexes can strengthen the spectral signals related to nitrogen through the combination of specific sensitive bands, effectively suppressing the influence of noise such as the soil background, atmospheric scattering and so on (Lee et al., 2025). In addition, machine learning algorithms can effectively reveal the complex nonlinear correlation between spectral features and nitrogen content by virtue of their powerful nonlinear fitting and feature mining capabilities (Li et al., 2025).

Despite significant progress in related research, obvious research gaps remain in nitrogen inversion for mountainous kiwifruit: (1) Most existing studies focus on field crops or plain orchards, and the synergistic interference mechanism of spectral signal distortion caused by mountain terrain undulation and canopy structure heterogeneity on inversion models has not been clearly elucidated; (2) As a vine fruit tree, kiwifruit has a unique canopy morphology (e.g., high porosity), and nitrogen inversion features and models developed for traditional herbaceous crops or arbor trees cannot be directly adapted; (3) There is a lack of systematic application of multi-feature fusion and ensemble learning in mountainous kiwifruit nitrogen inversion, leading to insufficient model adaptability to mountainous scenarios and room for improvement in inversion accuracy. Against this background, this study selected a mountainous kiwifruit orchard in Zhaotong, Yunnan Province as the study area, focusing on the critical nitrogen demand period (fruit expansion stage). Canopy high-resolution images were acquired via a UAV-mounted multispectral sensor, and ground-measured nitrogen data were collected synchronously. Core spectral parameters were extracted, and a univariate linear regression model and multiple machine learning models were constructed. Model performance was optimized using an ensemble learning strategy, ultimately achieving high-precision inversion and spatial distribution visualization of kiwifruit canopy nitrogen content. This study aims to provide a solid theoretical basis and practical technical support for precise fertilization and fine management of mountainous kiwifruit orchards.

## 2 Materials and methods

### 2.1 Overview of the study area

**Figure 1:**
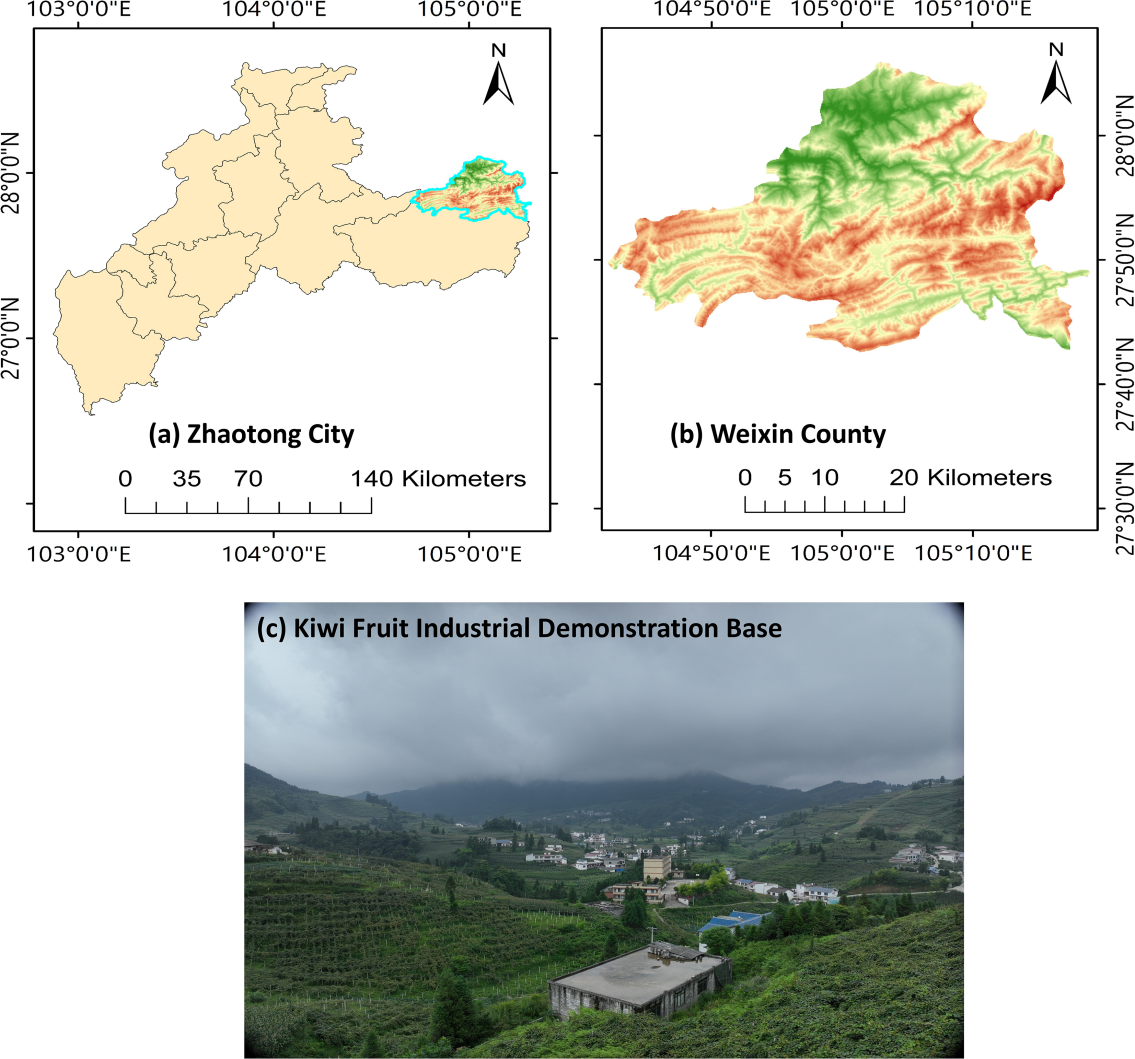
Schematic diagram of the geographic location of the study area. **(a)** Geographical location of Zhaotong City; **(b)** Geographical location of Weixin County; **(c)** Field landscape of the kiwifruit industry demonstration base.

This experiment was carried out at the kiwifruit industry demonstration base in Weixin County, Zhaotong City, Yunnan Province, China (104°54′56″E, 27°50′17″N, with an average elevation of 1131 m) (**Figure 1**). The region features a subtropical monsoon climate with favorable hydrothermal conditions for kiwifruit growth, with an average annual precipitation of ~1033 mm, an average annual temperature of 13.6 °C, and an average annual sunshine duration of 970.8 h.

The study area is mainly planted with “Guichang” kiwifruit on a large scale, with row spacing of 2 m. August is the fruit expansion stage of kiwifruit, which is a key point of plant growth and development, with strong nitrogen demand, and the supply of nitrogen directly affects the growth of fruits and nutrient accumulation, which provides an ideal window of time for the multispectral inversion of canopy nitrogen. Based on the actual planting pattern in the field, we formulated a test plot delineation plan, taking a single kiwifruit tree as the center, extending 1 m in all directions to delineate square test plots (2 m × 2 m, 4 m²), and finally determining 278 effective test plots, which provides a guarantee for the accurate matching between the UAV multispectral image acquisition and the ground-based nitrogen content measurement data.

### 2.2 Data acquisition

#### 2.2.1 UAV multispectral image acquisition

In this study, a DJI Mavic 3 Multispectral UAV was used as the data acquisition platform. The UAV integrates a 20-megapixel visible light camera and four 5-megapixel multispectral cameras, covering four core spectral bands with central wavelengths: green (550 nm), red (650 nm), red edge (730 nm), and near-infrared (NIR, 850 nm), and is equipped with an RTK real-time dynamic differential positioning module, which realizes centimeter-level spatial positioning and guarantees the spatial accuracy of the images. The DJI GS PRO professional ground station plans the “S” type automatic navigation route, and combined with the mountainous terrain characteristics of the study area, the terrain-following flight mode was enabled to maintain a constant relative altitude between the UAV and the canopy. The flight parameters were set as follows: flight altitude of 30 m, route speed of 2 m/s, heading overlap rate of 85%, side overlap rate of 70%, and multispectral camera lens pointing vertically at the canopy.

The image acquisition was conducted daily from 11:00 to 14:00 (Beijing Time), selecting clear, windless and cloudless weather conditions, when the sun’s altitude angle is suitable and the light conditions are stable, which minimizes shadow interference and temporal variations in spectral reflectance, ensuring the quality of spectral data.

#### 2.2.2 Ground-truthing nitrogen data collection

In order to reduce systematic errors and guarantee the accuracy of model inversion, ground sampling was synchronized with UAV image acquisition. Within the 278 experimental plots, three plants with uniform growth status (no pests or diseases) were randomly selected per plot, and five healthy, sun-exposed functional leaves from the middle-upper canopy of each plant were collected, with a total of 15 leaves collected in each plot for nitrogen content determination.

Leaf nitrogen content was measured using Yunyi Hengmei HM-YC handheld plant nutrient meter (Shandong Hengmei Electronic Technology Co., Ltd., Weifang, Shandong, China), and preheating, zero calibration and standard plate calibration were completed according to the instructions of the instrument before measurement. The five-point sampling method was adopted for each leaf: five evenly distributed points were measured in the middle region of the leaf (avoiding leaf veins), and the average value was taken as the nitrogen content of the leaf; the average value of the nitrogen content of all leaves in the plot was further calculated as the representative value of the canopy nitrogen content of the test plot. The coordinates and elevations of the sampling points were acquired synchronously by the UAV RTK system to ensure the spatial matching accuracy between the image pixels and the measured data.

#### 2.2.3 UAV data preprocessing

The preprocessing of the acquired multispectral images follows the standardized process of “correction, stitching, cropping, and information extraction”, with the following specific steps: (1) Geometric and radiometric correction: To address geometric distortions caused by wind speed and attitude changes during UAV flight, and radiometric distortions induced by terrain undulation and uneven illumination, geometric fine correction was performed using ENVI 5.6 software (Harris Geospatial Solutions, Broomfield, CO, USA) based on 15 ground control points (GCPs) distributed at the edge and interior of the study area. Absolute radiometric correction was completed using standard reflector data from the UAV, converting radiance values to surface reflectance to eliminate image biases and restore the true spectral information of the canopy. (2) Image stitching: Pix4Dmapper software (Pix4D SA, Lausanne, Switzerland) was used to stitch the corrected multispectral images, utilizing 85% of the overlapping area in the heading direction and 70% of the overlapping area in the side direction, and integrating the segmented image blocks through feature matching and fusion algorithms to generate a continuous and complete high-precision multispectral image of the study area with a spatial resolution of 0.05 m). (3) Image cropping: Using ENVI software, we cropped the image based on the boundary vector file of the study area, eliminated non-planting areas and edge interference areas, and obtained the target image containing only the kiwifruit canopy. (4) Spectral information extraction: The cropped images were imported into ArcGIS 10.8 software (Environmental Systems Research Institute, Redlands, CA, USA), and the “Extract Multi-Values to Points” tool was used to import the latitude and longitude coordinates of the central points of the 278 experimental plots, thereby accurately extracting the single-band reflectance values of each plot. This provided basic data for the subsequent calculation of vegetation indices.

### 2.3 Spectral variable construction

Based on the four core spectral bands (green, red, red edge, and near-infrared) of UAV multispectral images, combined with the spectral indices sensitive to crop nitrogen reported in existing studies, 25 spectral variables were screened and calculated. These variables included five single-band reflectances (blue, green, red, red edge, and near-infrared) and 20 vegetation indices (see **Table 1** for specific calculation formulas). Among them, the blue band was derived from the visible light camera mounted on the UAV: its blue channel information was extracted, and reflectance quantification was completed using Pix4Dmapper software. The reflectances of the other four single bands (green, red, red edge, and near-infrared) were directly acquired by the UAV’s multispectral camera. Based on the aforementioned spectral bands and the calculation formulas of vegetation indices, band operations were performed using Pix4Dmapper software to generate the spatial distribution maps of each spectral variable. All distribution maps were imported into ENVI 5.6 software, and the average value of each spectral variable in each experimental plot was calculated using the boundary vectors of the experimental plots as the characteristic value of the plot. Finally, a dataset of “278 experimental plots × 25 spectral variables” was constructed, containing a total of 6950 valid data points for the subsequent construction of nitrogen inversion models.

**Table 1:** Vegetation index selection.

Vegetation index	Formula	References
Normalized Difference Vegetation Index(NDVI)	$\frac{\text{RNIR}-\text{RRed}}{\text{RNIR}+\text{RRed}}$	(Li et al., 2021)
Ratio Vegetation Index(RVI)	$\frac{\text{RNIR}}{\text{RRed}}$	(Li et al., 2023)
Difference Vegetation Index(DVI)	RNIR−RRed	(Liu et al., 2021)
Green Normalized Difference Vegetation Index(GNDVI)	$\frac{\text{RNIR}-\text{RGreen}}{\text{RNIR}+\text{RGreen}}$	(Liu et al., 2022)
Optimized Soil-Adjusted Vegetation Index(OSAVI)	$\frac{\text{RNIR}-\text{RRed}}{\text{RNIR}+\text{RRed}+0.16}$	(Ma et al., 2019)
Soil-Adjusted Vegetation Index(SAVI)	$1.5\times \frac{\text{RNIR}-\text{RRed}}{\text{RNIR}+\text{RRed}+0.5}$	(Mahmud et al., 2023)
Renormalized Difference Vegetation Index(RDVI)	$\sqrt{\left(\text{RNIR}-\text{RRed}\right)\times \left(\text{RNIR}+\text{RRed}\right)}$	(Mao et al., 2020)
Normalized Difference Red Edge Index(NDRE)	$\frac{\text{RNIR}-{\text{RRed}}_{\text{edge}}}{\text{RNIR}+{\text{RRed}}_{\text{edge}}}$	(Morales-Gallegos et al., 2023; Nogueira et al., 2025)
Modified Simple Ratio Index(MSR)	$\frac{\sqrt{\frac{\text{RNIR}}{\text{RRed}}}-1}{\sqrt{\frac{\text{RNIR}}{\text{RRed}}}+1}$	(Pandey et al., 2022)
Green-Red Normalized Index(GRVI)	$\frac{\text{RNIR}}{\text{RGreen}}$	(Pei et al., 2023)
Modified Soil-Adjusted Vegetation Index(MSAVI)	$\frac{2\times \text{RNIR}+1-\sqrt{{\left(2\times \text{RNIR}+1\right)}^{2}-8\times \left(\text{RNIR}-\text{RRed}\right)}}{2}$	(Qiao et al., 2022)
Triangular Vegetation Index(TVI)	$0.5\times \left[120\times \left(\text{RNIR}-{\text{RRed}}_{\text{edge}}\right)-200\times \left(\text{RRed}-{\text{RRed}}_{\text{edge}}\right)\right]$	(Qin et al., 2025)
Transformed Chlorophyll Absorption in Reflectance Index(TCARI)	$3\times \left[\left({\text{RRed}}_{\text{edge}}-\text{RRed}\right)-0.2\times \left({\text{RRed}}_{\text{edge}}-\text{RGreen}\right)\times \frac{{\text{RRed}}_{\text{edge}}}{\text{RRed}}\right]$	(Sellami et al., 2022)
Leaf Chlorophyll Index(LCI)	$\frac{\text{RNIR}-{\text{RRed}}_{\text{edge}}}{\text{RNIR}+\text{RRed}}$	(Shao et al., 2023)
Normalized Difference Water Index(NDWI)	$\frac{\text{RGreen}-\text{RNIR}}{\text{RGreen}+\text{RNIR}}$	(Stefaniak and Łata, 2023)
Nitrogen Reflectance Index(NRI)	$\frac{\text{RGreen}-\text{RRed}}{\text{RGreen}+\text{RRed}}$	(Sun et al., 2024)
Non-Linear Vegetation Index(NLI)	$\frac{{\left(\text{RNIR}-\text{RRed}\right)}^{2}}{{\left(\text{RNIR}+\text{RRed}\right)}^{2}}$	(Zhen et al., 2020)
Modified Non-Linear Vegetation Index(MNLI)	$1.5\times \frac{{\text{RNIR}}^{2}-{\text{RGreen}}^{2}}{{\text{RNIR}}^{2}+{\text{RGreen}}^{2}+0.5}$	(Yu et al., 2025)
Modified Triangular Chlorophyll Index(MTCI)	$\frac{\text{RNIR}-{\text{RRed}}_{\text{edge}}}{{\text{RRed}}_{\text{edge}}-\text{RRed}}$	(Zhao et al., 2025)
Chlorophyll Absorption in Reflectance Index(CARI)	$\left({\text{RRed}}_{\text{edge}}-\text{RRed}\right)-2.0\times \left({\text{RRed}}_{\text{edge}}-\text{RGreen}\right)\times \frac{{\text{RRed}}_{\text{edge}}}{\text{RRed}}$	(Zheng et al., 2022)

In the formula, RGreen, RRed, RRededge and RNIR denote Green (green band), Red(red band), Rededge (red edge band), and NIR (near infrared band) reflectance.

### 2.4 Single model modeling

#### 2.4.1 Linear regression

LR is a classical statistical modeling method. Its core principle is to establish a linear mapping relationship between predictor variables (spectral variables) and the response variable (canopy nitrogen content) by minimizing the sum of squared residuals between predicted and measured values. This method features a simple structure, clear physical significance, and strong interpretability, making it suitable for preliminary linear correlation analysis between spectral variables and nitrogen content.

#### 2.4.2 Backward stepwise regression

BSR is a core implementation strategy of Multiple Stepwise Regression (MSR). It adopts a backward elimination approach for modeling: an initial model is constructed using all 25 candidate spectral variables, and redundant variables with non-significant regression coefficients are gradually eliminated based on the p-value significance test (α = 0.05) until all retained features meet the statistical significance criterion. Ultimately, the optimal multiple linear regression equation is established. This method can effectively simplify the model structure, mitigate multicollinearity in high-dimensional spectral data, and enhance modeling efficiency and the interpretability of results.

#### 2.4.3 Support vector machine regression

SVR is a classic nonlinear algorithm in machine learning. It primarily maps the original low-dimensional feature space composed of spectral variables into a high-dimensional feature space using the radial basis function (RBF) kernel, thereby transforming the nonlinear relationship between spectral variables and nitrogen content into a solvable linear regression problem. Meanwhile, it introduces the penalty coefficient C and kernel coefficient γ to regulate model complexity and generalization ability, exhibiting prominent advantages in modeling with high-dimensional data, small samples, and nonlinear relationships.

#### 2.4.4 Random forest regression

RFR is a nonlinear regression algorithm rooted in the ensemble learning paradigm. It constructs an ensemble model consisting of multiple decision trees and adopts Bootstrap resampling and random feature selection strategies, which effectively reduces the overfitting risk of a single decision tree and enhances the model’s ability to capture intrinsic patterns in complex spectral data as well as its generalization stability. This method does not require strict normal distribution assumptions for the input data. It can efficiently handle redundant information and multicollinearity issues in high-dimensional spectral data, while exhibiting strong robustness to noise interference.

#### 2.4.5 Partial least squares regression

PLSR is a multivariate statistical method that balances dimensionality reduction and regression analysis. Its core is to extract latent variables (principal components) that are mutually correlated with both predictor variables (spectral variables) and the response variable (canopy nitrogen content), while maximizing the variance of the explanatory variables. Based on these latent variables, a regression model between principal components and canopy nitrogen content is established. This method can effectively address the multicollinearity problem in high-dimensional spectral data and has low requirements on sample size, making it suitable for the nitrogen inversion task with limited experimental plots (278 plots).

### 2.5 Ensemble learning modeling

#### 2.5.1 Bagging ensemble learning

Bagging is a parallel ensemble learning method based on Bootstrap resampling. Its core mechanism involves randomly resampling the original dataset with replacement 100 times to generate 100 differentiated sub-datasets, training independent base learners on each sub-dataset, and finally outputting the integrated prediction results via a mean fusion strategy. This method is well-suited for multispectral high-dimensional feature data and scenarios where a single model tends to overfit. By leveraging the diversity of base learners, it reduces model variance, mitigates the limitations of individual models, and significantly enhances inversion stability and anti-interference capability. In this study, the well-trained single model PLSR was directly employed as the core base learner of Bagging. Based on the excellent fitting performance and generalization stability of this optimal single model, the ensemble modeling advantages of Bagging were maximized.

#### 2.5.2 Boosting ensemble learning

Boosting is a sequential ensemble learning method centered on error optimization. Its core process involves sequentially and iteratively training 100 base learners: sample weights are adjusted according to the prediction errors of the previous training round (assigning higher weights to mispredicted samples) to enhance the model’s ability to capture key information, and the outputs of all base learners are finally fused through a weighted fusion strategy. This method exhibits significant advantages in scenarios involving weak learners, nonlinear relationships, and small-sample data, as it can effectively correct prediction bias and explore the potential patterns inherent in the data. In this study, a Boosting ensemble model was constructed based on GradientBoostingRegressor. The model was directly established using the GradientBoostingRegressor base learner optimized via 5-fold cross-validation. This allowed the base learner to maintain the properties of a weak learner, which not only avoided model overfitting but also preserved its error correction ability.

#### 2.5.3 Stacking ensemble learning

Stacking is a fusion-based ensemble learning method relying on hierarchical modeling. In this study, a two-layer modeling framework was constructed for it, which integrates the advantages of multiple models based on the complementarity of their inherent modeling mechanisms and fully extracts both linear and nonlinear characteristics embedded in spectral data. This approach is particularly suitable for scenarios involving multi-model complementarity, high-dimensional heterogeneous spectral data processing, and high-precision nitrogen inversion tasks. Based on the two-dimensional complementary principle (linear–nonlinear and dimensionality-reduction–pure regression), the first layer of the model directly adopts five optimized single models (LR, SVR, BSR, RFR, and PLSR) as base learners, with clear functional division: linear algorithms (LR, BSR) mine linear spectral features from the perspectives of baseline fitting and stepwise feature screening (to mitigate multicollinearity), while nonlinear algorithms (SVR, RFR) capture complex nonlinear relationships between spectral variables and nitrogen content; PLSR, as a dimensionality-reduction regression model, complements the limitations of pure linear/nonlinear models in handling high-dimensional spectral data. Each base learner outputs independent prediction results after full optimization. The second layer takes the prediction results of all base learners as new high-level features and feeds them into the meta-learner constructed by LR. The fusion of base-model predictions is completed via 5-fold cross-validation to ensure the robustness of the integrated results. This hierarchical modeling strategy effectively leverages the complementary information from each optimized base learner, significantly improving the model’s adaptability to the complex scenario of nitrogen inversion in mountainous kiwifruit.

### 2.6 Model evaluation indicators and validation methods

#### 2.6.1 Evaluation indicators

Pearson’s correlation coefficient (r), coefficient of determination (R2), root mean squared error (RMSE), and relative percent difference (RPD) were selected as the model performance evaluation indicators. The core function of each indicator is as follows: r: Quantifies the strength of the linear correlation between the independent variable and the dependent variable; R2: Reflects the proportion of the variance in the dependent variable explained by the model (range: 0-1); RMSE: Quantifies the prediction error and characterizes the prediction accuracy; RPD: Evaluates the model’s generalization performance with the following reference criteria: RPD > 2.0 (excellent), 1.4 ≤ RPD ≤2.0 (good), RPD< 1.4 (poor).

The specific formulas for each indicator are as follows (Equations 1–4):

(1) $$r=\frac{\sum_{i=1}^{N}\left(x_{i}-\bar{x}\right)\left(y_{i}-\bar{y}\right)}{\sqrt{\sum_{i=1}^{N}{\left(x_{i}-\bar{x}\right)}^{2}}\cdot \sqrt{\sum_{i=1}^{N}{\left(y_{i}-\bar{y}\right)}^{2}}}$$

(2) $$R^{2}=1-\frac{\sum_{i=1}^{N}{\left(y_{i}-{\hat{y}}_{i}\right)}^{2}}{\sum_{i=1}^{N}{\left(y_{i}-\bar{y}\right)}^{2}}$$

(3) $$RMSE=\sqrt{\frac{1}{N}\sum_{i=1}^{N}{\left(y_{i}-{\hat{y}}_{i}\right)}^{2}}$$

(4) $$RPD=\frac{SD}{RMSE}=\frac{\sqrt{\frac{1}{N-1}\sum_{i=1}^{N}{\left(y_{i}-\bar{y}\right)}^{2}}}{\sqrt{\frac{1}{N}\sum_{i=1}^{N}{\left(y_{i}-{\hat{y}}_{i}\right)}^{2}}}$$

Note: ${\hat{y}}_{i}$ is the predicted value of nitrogen content for the ith sample, ${\text{y}}_{\text{i}}$ is the corresponding measured value, $\bar{y}$ is the average of the measured values of all samples, ${\text{x}}_{\text{i}}$ is the value of the independent variable (spectral variable) for the ith sample, $\bar{x}$ is the mean value of the independent variable, N is the number of samples, and SD is the standard deviation of the measured values of the samples.

#### 2.6.2 Validation methods

To reduce the risk of model overfitting, this study adopts 5-fold cross-validation. The dataset is divided into 5 equal-sized subsets based on the principle of stratified sampling, followed by 5 iterative training and validation processes. In each iteration, one subset is designated as the validation set, and the remaining four are combined into the training set, ensuring that all data participate in both training and validation. The final model performance is determined by averaging the validation results of the 5 iterations, enabling a comprehensive and objective evaluation of the model’s generalization ability.

### 2.7 Hyperparameter optimization

To ensure the stability and generalization ability of the core predictive models, hyperparameter optimization was performed for models with complex tunable hyperparameters, including SVR, RFR, and Boosting ensemble model. The GridSearchCV algorithm was adopted, combined with 5-fold cross-validation to avoid overfitting during the parameter tuning process. The specific parameter search ranges were set as follows: (1) For the SVR model, the penalty coefficient C was set to [0.1, 1, 10, 100], and the kernel coefficient γ was set to [0.001, 0.01, 0.1, 1]; (2) For the RFR model, the maximum tree depth max_depth was set to [5, 10, 15], and the number of decision trees n_estimators was set to [50, 100, 150]; (3) For the Boosting model, the learning rate was set to [0.01, 0.1, 0.2], max_depth was set to [3, 5, 7], and n_estimators was set to [100, 200, 300]. No additional hyperparameter tuning was conducted for models with inherently simple structures or fixed algorithmic frameworks in this study: (1) LR and BSR adopted default configurations directly due to their simple linear structures without complex hyperparameters requiring optimization; (2) The core performance of PLSR is determined by the number of latent variables, which has been optimized through cross-validation within the algorithm itself; (3) The core mechanism of the Bagging ensemble model relies on Bootstrap resampling and mean fusion. In this study, the number of resampling times was fixed at 100, and the pre-optimized PLSR model was used as the base learner, eliminating the need for additional tuning of the Bagging framework; (4) The Stacking ensemble model was constructed based on the principle of “directly calling pre-optimized base learners”, and its linear meta-learner (LR) is sufficient to achieve complementary fusion of prediction results. Therefore, no tuning was performed for the Stacking framework and the meta-learner.

### 2.8 SHapley Additive exPlanations

Interpretability is a key prerequisite for the practical application of machine learning models in agricultural remote sensing. It can clarify the dominant feature factors affecting the model’s prediction results and provide support for verifying model reliability and guiding field management decisions. As a mainstream interpretability analysis tool, SHAP (SHapley Additive exPlanations) offers advantages including strong model independence, a solid theoretical foundation (rooted in game theory), and excellent visualization effects. It can intuitively quantify and present the contribution of each feature in any model to the prediction results, as well as the direction of each feature’s influence.

Its core formula is shown in Equation 5.

(5) $$\phi_{i}=\sum_{S\subseteq F\setminus \left\{i\right\}}\frac{\left|S\right|!\;\left(M-\left|S\right|-1\right)!}{M!}\left[f\left(S\cup \left\{i\right\}\right)-f\left(S\right)\right]$$

Note: $\left(\phi_{\text{i}}\right)$ is the SHAP value of the ith feature (characterizing the net contribution of the feature to the prediction result); F is the full set of input features to the model; S is any subset of F that does not contain the ith feature; M is the total number of input features; f(S) is the predicted value of the model when only a subset of S is input; and f(S∪​{i}) is the predicted value of the model when a subset of S is input with the ith feature.

## 3 Results

### 3.1 Pearson correlation analysis

**Figure 2:**
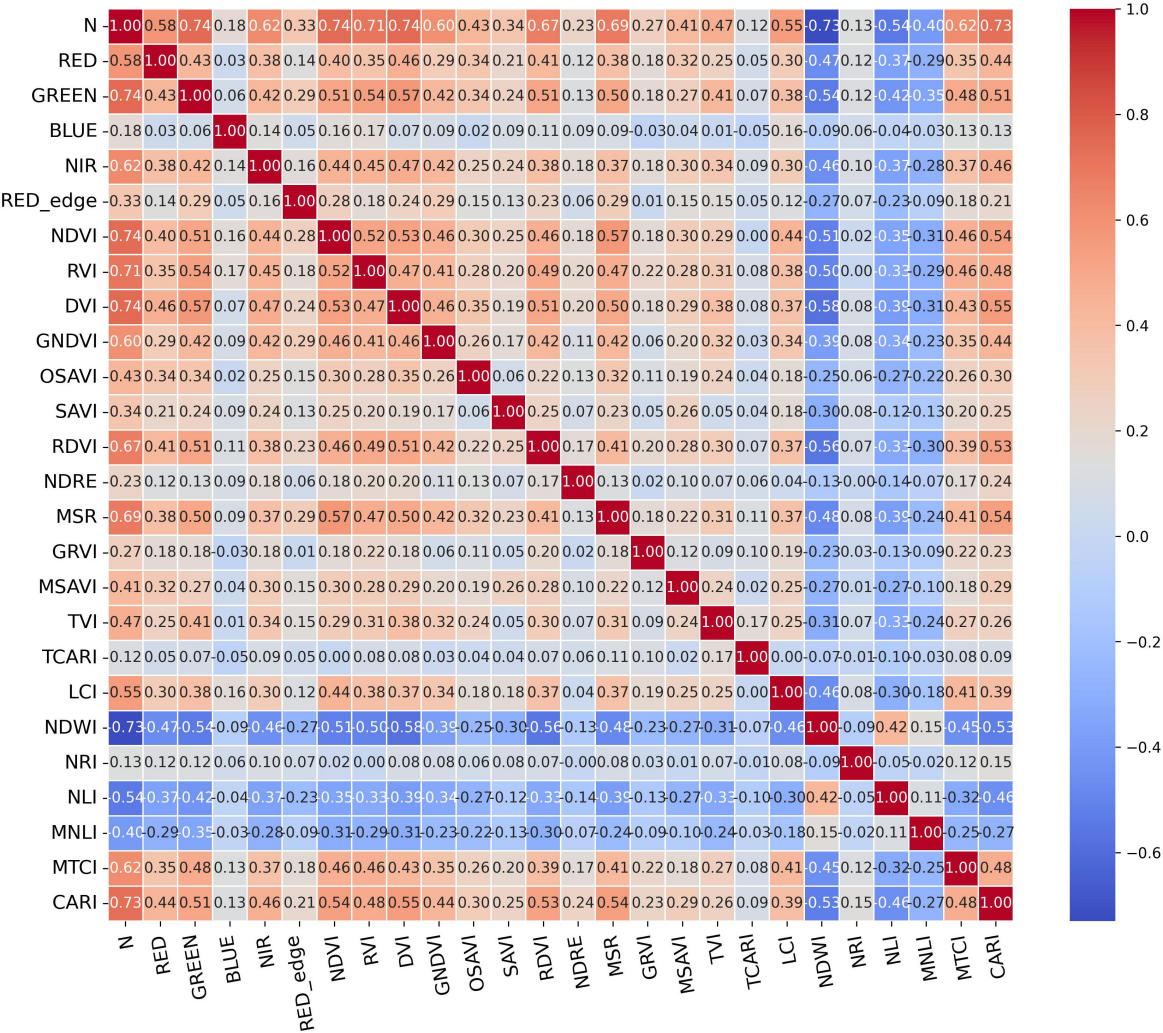
Heat map of correlation analysis matrix.

To screen spectral variables that are sensitive and statistically reliable for kiwifruit canopy nitrogen (N) content, reduce the complexity of subsequent modeling, and improve inversion accuracy, this study employed Pearson’s correlation analysis to quantify the strength and direction of linear associations between 25 spectral variables (5 single-band reflectances and 20 vegetation indices) and canopy N content. The statistical validity of these associations was verified using p-values from t-tests with significance levels of α = 0.05 and α = 0.01 (**Figure 2**).

The correlation analysis results showed the following: (1) Among single-band features, the GREEN band exhibited a highly significant correlation with nitrogen content (p< 0.01) with an absolute correlation coefficient |r| > 0.7; the NIR band showed a significant correlation with nitrogen content (p ≤ 0.05) with |r| > 0.6; (2) Among vegetation indices, NDVI, RVI, DVI, NDWI, and CARI displayed highly significant correlations with nitrogen content (p< 0.01, |r| > 0.7); GNDVI, RDVI, MSR, and MTCI showed significant correlations with nitrogen content (p ≤ 0.05, |r| > 0.6); (3) The correlations between the remaining spectral variables and nitrogen content did not reach statistical significance (p > 0.05).

### 3.2 Univariate linear regression inverse models

To investigate the independent predictive ability of the six highly significantly correlated features (GREEN, NDVI, RVI, DVI, NDWI, CARI) screened for kiwifruit canopy N content, this study constructed individual LR models for each single feature, and analyzed the hierarchical sensitivity of spectral variables in characterizing nitrogen content, with test set performance as the core evaluation criterion (**Table 2**).

**Table 2:** Test set results of univariate LR models.

Spectral variable	Regression equation	R^2^	RMSE	RPD
GREEN	Y=59736.69x + 4.90	0.59	1.07	1.57
NDVI	Y=57.76x - 33.64	0.48	1.21	1.39
RVI	Y=0.50x + 8.42	0.42	1.27	1.33
DVI	Y=5985.52x + 6.93	0.58	1.08	1.56
NDWI	Y=-53.75x - 24.65	0.58	1.08	1.56
CARI	Y=48567.97x - 19.52	0.51	1.17	1.44

The modeling results indicate significant differences in inversion accuracy among different features. Among these, the GREEN band exhibited the best performance, followed by DVI and NDWI with a test set R2 of 0.58. In contrast, RVI performed the worst and was significantly less sensitive to nitrogen content than single-band features. Overall, the test set R2 of all univariate LR models ranged from 0.42 to 0.59, which is insufficient to meet the high-accuracy requirements for field precision management. The core limitation may be that a single spectral feature can only reflect the linear correlation between nitrogen content and a specific spectral dimension, and cannot fully capture the complex variations in nitrogen content driven by soil fertility, field light conditions, and plant growth status. Thus, inversion performance needs to be further improved through multi-feature integration.

### 3.3 Multivariate modeling

To address the insufficient inversion accuracy of the LR model caused by a single feature, four multivariate models (BSR, PLSR, RFR, and SVR) were established with all 25 spectral variables as inputs. Their generalization ability was evaluated via 5-fold cross-validation, and the optimal test set performance of each model is summarized in **Table 3**.

**Table 3:** Optimal test set results of multivariate models.

Model	R^2^	RMSE	RPD
LR	0.59	1.06	1.57
BSR	0.85	0.58	2.67
SVR	0.82	0.65	1.97
RFR	0.83	0.69	2.44
PLSR	0.86	0.63	2.68

The BSR model quantifies variable contributions through endogenous feature screening, eliminating 13 redundant variables from the 25 spectral variables and ultimately retaining 12 core features (including GREEN and NIR single bands, as well as NDVI, RVI, RED, DVI, GNDVI, MSR, NDWI, MNLI, MTCI, and CARI vegetation indices). This model exhibited the best test set performance, achieving a balance between fitting accuracy and generalization stability. The PLSR model mitigates multicollinearity through feature dimensionality reduction; the optimal number of principal components was determined to be 1 via 5-fold cross-validation. Its error metrics are close to those of the BSR model without obvious overfitting, highlighting its suitability for multi-feature collinearity scenarios. The RFR model is constructed based on the Bagging strategy and optimized using GridSearchCV (5-fold cross-validation) to determine the optimal hyperparameters (max_depth= 15, n_estimators= 150). The model achieved extremely high training set fitting accuracy (R2 = 0.97, RMSE = 0.24), but its test set generalization ability declined. This is presumably due to overfitting caused by the multi-decision tree overlearning noise in the training set. For the SVR model, optimal hyperparameters (C = 100, γ= 0.1) were tuned with an average cross-validation score of 0.723, achieving a training set performance of R2 = 0.91 and RMSE = 0.50. However, the test set showed significant overfitting, reflecting the model’s hyperparameter sensitivity to high-dimensional features and its limitations in generalization.

Comprehensive comparisons indicated that the inversion accuracies of both the BSR and PLSR models reached a robust level (R2> 0.85). Among these, PLSR was the optimal single model, with higher fitting accuracy and smaller errors; the RFR model ranked second due to overfitting; SVR exhibited the worst generalization ability; and there was a significant performance gap between the univariate LR model and the multivariate models. These results confirm that multi-feature integration combined with scientific feature screening is the key to improving nitrogen inversion accuracy.

### 3.4 Comparison of ensemble ML models

To further improve the accuracy and generalization stability of kiwifruit canopy nitrogen inversion, the five constructed single models were used as base learners, and three ensemble strategies (Bagging, Boosting, and Stacking) were adopted to build ensemble models. All ensemble models took all 25 spectral variables as inputs, and the dataset was randomly split into training and test sets at an 8:2 ratio (repeated three times to reduce random bias). Model parameters were optimized and performance was evaluated via 5-fold cross-validation, with the optimal test set results summarized in **Table 4**.

**Table 4:** Performance comparison of ensemble learning models.

Model	R^2^	RMSE	RPD
Bagging	0.86	0.58	2.69
Boosting	0.89	0.50	2.99
Stacking	0.87	0.56	2.75

The Boosting ensemble model achieved the best test set performance, with a good fit between predicted and measured values. This is attributed to its iterative weighting mechanism, which strengthens the capture of key information and improves fitting accuracy. The Bagging ensemble model exhibited extremely high training set fitting accuracy (R2 = 0.90), but its test set performance declined significantly. This is presumably because the algorithm is not optimized for outliers, and the mean fusion of predictions from multiple base learners fails to completely avoid noise interference in the training set, thereby limiting its generalization ability. The Stacking ensemble model exhibited robust test set performance, effectively integrating the complementary strengths of linear and nonlinear models through the hierarchical modeling strategy, and improving the adaptability to complex data patterns.

Comprehensive comparisons of the ensemble models show that the Boosting model achieved the highest R2 and the smallest RMSE in the test set, exhibiting the best fitting performance; the Stacking model ranked second; and the Bagging model performed relatively weakly due to insufficient generalization stability. All three types of ensemble models outperformed the optimal single model (PLSR), highlighting the significant advantage of ensemble learning in improving inversion performance. Considering the complex topography of mountain kiwifruit orchards, where spectral signals are susceptible to interference from terrain undulation and canopy heterogeneity, the Boosting model strengthens key information capture through a serial iterative weighting mechanism. This gives it greater advantages in bias correction and anti-interference adaptability, and it is ultimately identified as the optimal model for kiwifruit canopy nitrogen inversion in this study. It can provide reliable technical support for the accurate nitrogen inversion of mountain kiwifruit.

### 3.5 SHAP interpretability analysis

To elucidate the driving mechanism of nitrogen inversion in the optimal Boosting ensemble model, verify the scientific validity of spectral feature selection, and break through the “black box” limitation of machine learning models, this study employs the SHAP method to quantitatively dissect the contribution strength and influence patterns of spectral variables on inversion results from two dimensions: global quantification of feature importance and single-feature action mechanism, thereby systematically revealing the model’s prediction logic.

#### 3.5.1 Global quantification of feature importance

**Figure 3:**
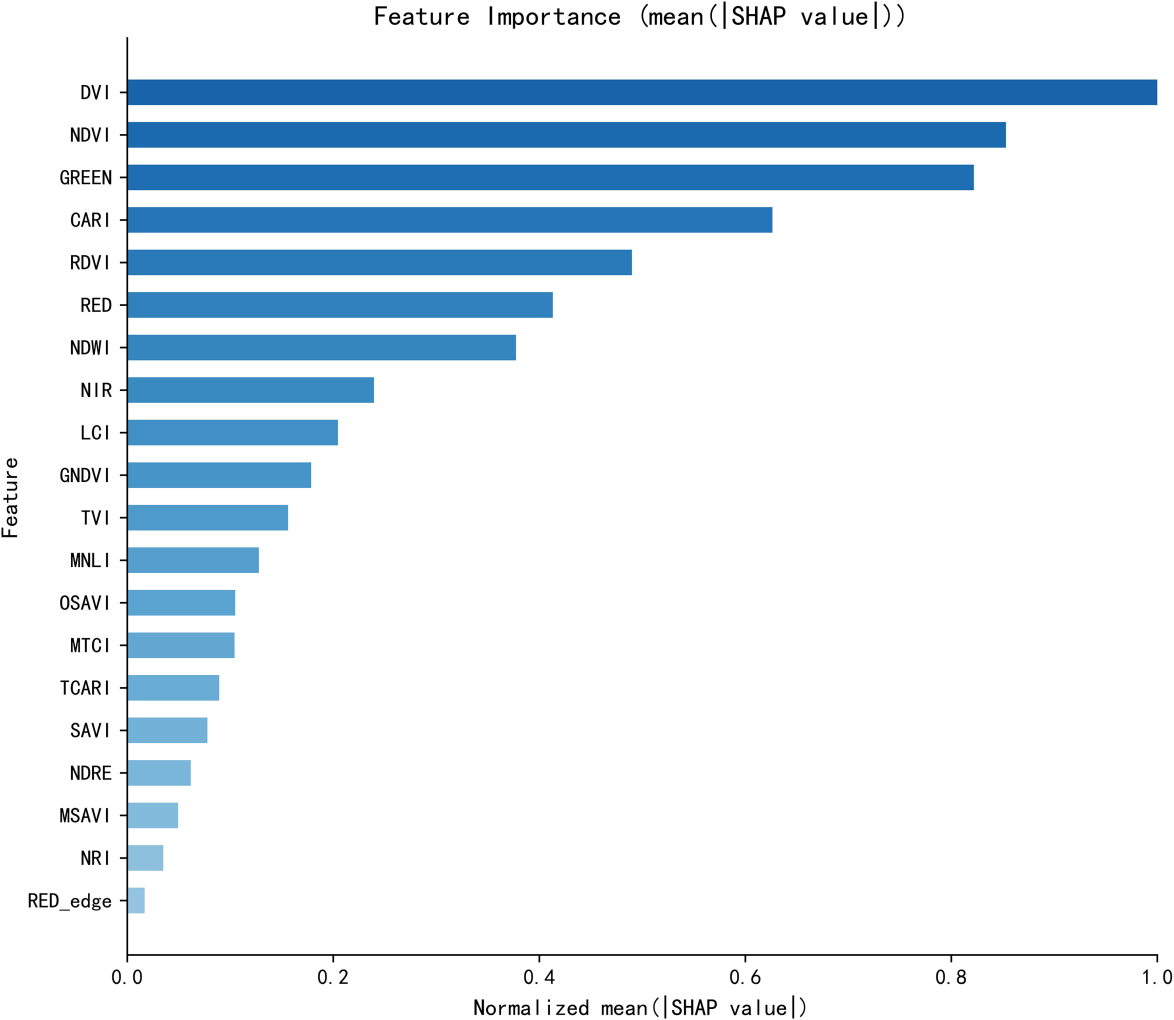
Ranking of SHAP feature importance. The x-axis is the normalized mean absolute SHAP value, characterizing the strength of the net contribution of each spectral variable to the model predictions; the y-axis is the spectral variable input to the model.

SHAP feature importance is quantified using the mean absolute SHAP value as the core metric, which intuitively characterizes the strength of the net contribution of each spectral variable to the model’s prediction results. The corresponding analysis results are presented in **Figure 3**.

As shown in **Figure 3**, the importance of core spectral variables exhibits a gradient distribution: DVI is the core feature with the strongest contribution to model predictions, with a mean absolute SHAP value close to 1.0; the contribution strengths of NDVI and the GREEN band decrease sequentially, and both are classified as high-importance features; the contribution strength of CARI ranks fourth, making it another core driving feature. The cumulative contribution of the top six high-importance features (DVI, NDVI, GREEN, CARI, RDVI, RED) accounts for 69.62%, which are the key information carriers for the model to achieve nitrogen inversion.

This result, on the one hand, verifies the signal enhancement effect of band combination indices (e.g., DVI and CARI) on nitrogen-related spectral signals; on the other hand, it clarifies the core position of the GREEN band as a nitrogen-sensitive feature. This further supports the scientific validity and rationality of the feature screening strategy adopted in this study.

#### 3.5.2 Mechanisms of action and patterns of influence of single features

**Figure 4:**
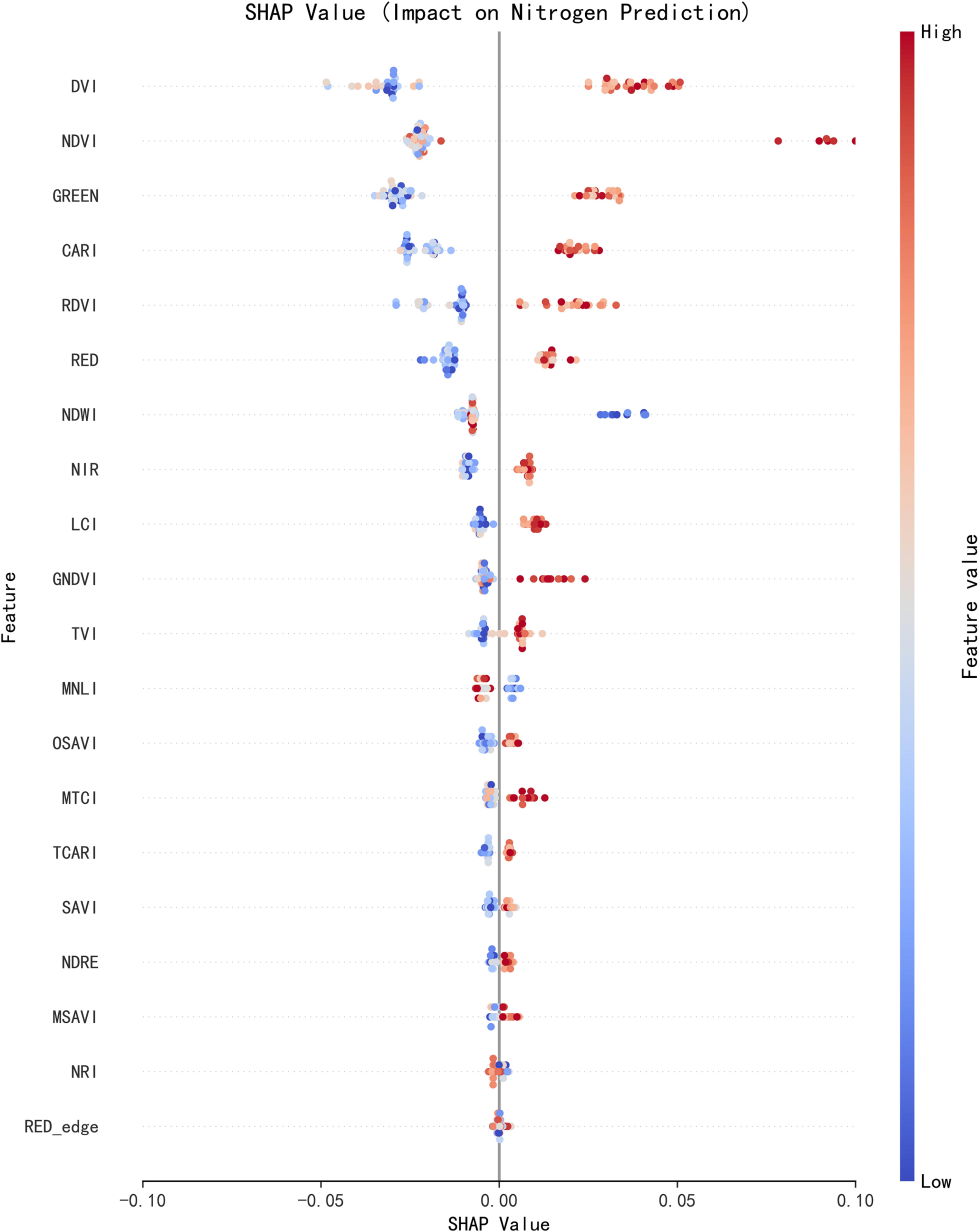
SHAP dependency graph for core features. The x-axis is the SHAP value (positive values promote higher nitrogen predictions and negative values suppress them); the y-axis is the spectral variable entered into the model; the color gradient represents the normalized value of the spectral variable.

The SHAP dependence plot (**Figure 4**) visualizes the quantitative correlation between core features and nitrogen predicted values, and its trend is highly consistent with spectroscopic principles and crop physiological mechanisms.

DVI is the primary driving feature for nitrogen prediction, with its SHAP value showing a significantly positive correlation with its own standardized value. The higher the DVI value, the stronger its contribution to the nitrogen predicted value, enabling it to directly characterize the level of nitrogen accumulation in plants. NDVI ranks second in contribution strength, and its SHAP value is also positively correlated with its standardized value, with concentrated data distribution, exerting a stable and reliable impact on prediction results. This is consistent with the core characteristic of NDVI, which can effectively reflect the chlorophyll content and nitrogen nutritional status of vegetation.

The GREEN band is a core negatively sensitive feature for nitrogen prediction, with its SHAP value showing a significantly negative correlation with its normalized value. As the reflectance of this band increases, the nitrogen predicted value decreases. This pattern is highly consistent with crop spectral physiological mechanisms: when nitrogen is sufficient, plant chlorophyll content increases, significantly enhancing the absorption capacity of green light, which in turn leads to a decrease in reflectance. This result further confirms the physiological basis for this band to serve as a nitrogen-sensitive feature.

CARI and RDVI are important auxiliary features. The SHAP value of CARI is positively correlated with the increase of its standardized value; this index effectively mitigates the interference of soil bareness and canopy shading in mountainous areas by integrating difference information from the RED edge, GREEN, and RED bands. The SHAP values of RDVI are concentrated in distribution, exerting a mild moderating effect on prediction results. Together, these two features can effectively supplement the information gaps of DVI, NDVI, and the GREEN band, reducing the model’s dependence on a single feature.

The SHAP values of classical vegetation indices (e.g., NDWI) are positively correlated with nitrogen predicted values, but their contribution strength is low. The core reason is that the design of these indices is not fully adapted to mountainous scenarios and kiwifruit vine characteristics: they are more sensitive to spectral variability induced by mountainous terrain, and the canopy porosity heterogeneity formed by kiwifruit branching and leaf growth further interferes with the stability of spectral signals. These factors collectively result in their limited contribution to nitrogen inversion.

### 3.6 Inverse thermogram of nitrogen content

**Figure 5:**
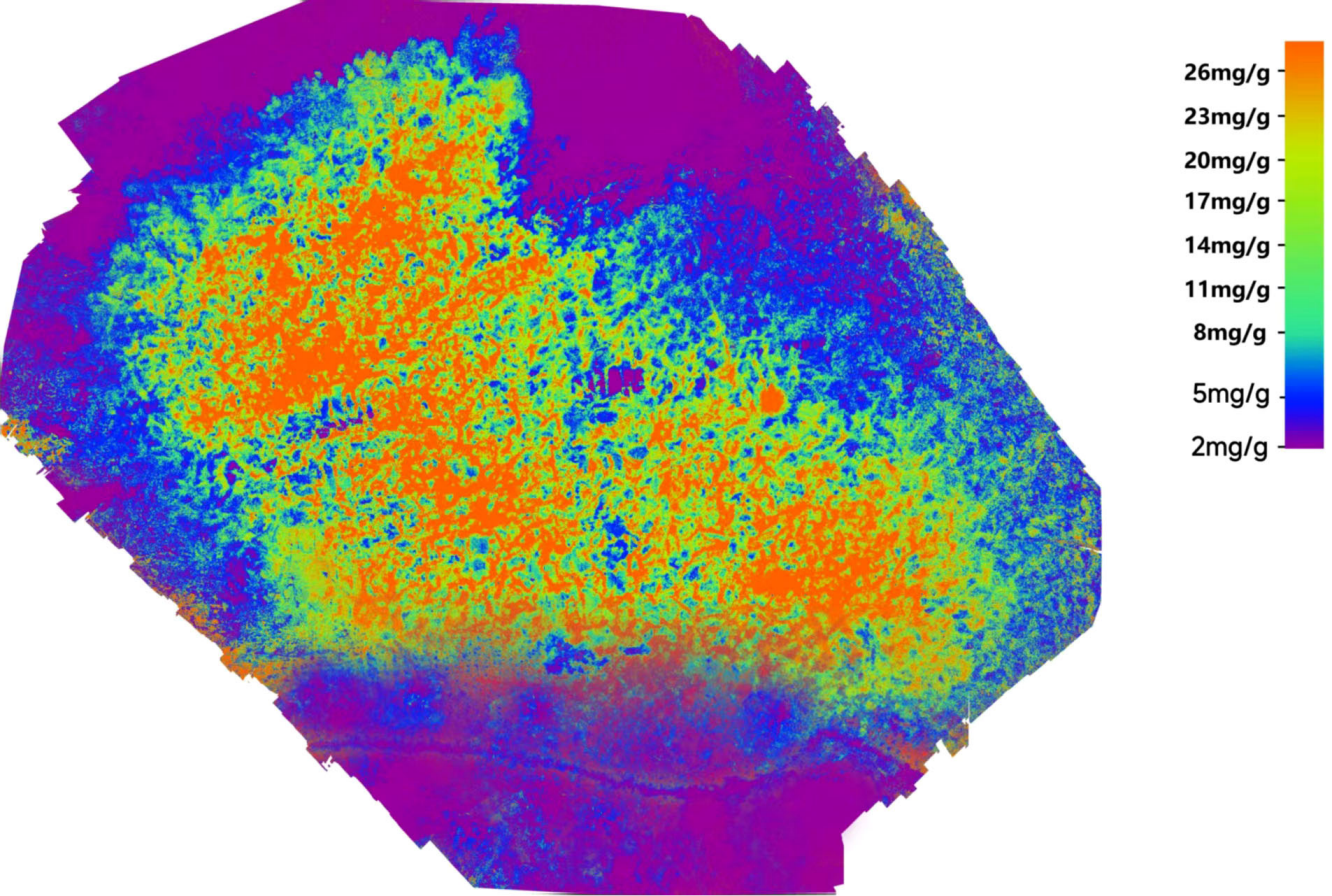
Nitrogen content inversion heatmap.

Based on the model performance comparison results, the Boosting ensemble model was identified as the optimal model for kiwifruit canopy nitrogen inversion. To intuitively characterize the spatial distribution of nitrogen in the study area and support field precision management, this model was used to generate a canopy nitrogen content heatmap of the experimental area (**Figure 5**). The heatmap quantifies nitrogen differences through color gradients, realizing the visual expression of inversion results and providing a technical basis for targeted regulation.

In the heatmap, purple areas represent non-kiwifruit planting regions, and blue areas indicate canopy shadow regions. In the core kiwifruit canopy region, dark orange areas denote nitrogen-enriched zones (≥26 mg/g), where sufficient canopy nitrogen supply fully meets the nitrogen demand for plant growth and fruit development during the fruit expansion stage. Cyan areas represent nitrogen-deficient zones (<8 mg/g), in which nitrogen content is far below the optimal level. This deficiency is presumed to be jointly affected by multiple factors, including the uneven water and nutrient migration caused by topographic relief in mountainous orchards, spatial heterogeneity in field fertilization practices, and background heterogeneity in soil fertility. As the study area was in the critical fruit expansion stage with high nitrogen demand, long-term nitrogen deficiency would not only inhibit vegetative growth such as leaf and branch development but also directly impede fruit expansion and nutrient accumulation, thereby significantly reducing single fruit weight and internal fruit quality. The transitional color regions (8–26 mg/g) are classified as medium-nitrogen zones, which are uniformly distributed across the study area. Canopy nitrogen content in these zones is at an appropriate level for normal growth, satisfying the basic nitrogen requirements for plant development and fruit expansion during the critical stage, and represents the baseline nutritional status of kiwifruit canopy nitrogen in the study area.

Based on the above spatial distribution patterns of nitrogen and the characteristics of the mountainous orchard environment in the study area, targeted precision management strategies for kiwifruit canopy nitrogen were proposed. For nitrogen-deficient zones, the core causes of nitrogen deficiency should first be identified through topographic investigation and soil sampling analysis. Then, in view of the topographic features such as upper slopes and steep slopes where water and nutrients are prone to loss, a precision nitrogen supplementation scheme based on water and fertilizer integration should be formulated. Slow-release nitrogen fertilizers applied via drip irrigation are preferred to reduce nitrogen loss while rapidly replenishing nitrogen for the fruit expansion stage. For nitrogen-enriched zones, the application rate of nitrogen fertilizer should be appropriately reduced, and phosphorus, potassium, and medium and trace element fertilizers should be increased to regulate the balance between vegetative and reproductive growth, avoiding excessive vegetative growth caused by over-nitrogen and reducing the risk of agricultural non-point source pollution from nitrogen leaching. For nitrogen-enriched plots in downslope and low-lying areas prone to water and nutrient accumulation, field drainage facilities should be added simultaneously to improve soil aeration and promote efficient nitrogen utilization. For the entire orchard, the irrigation regime and planting density should be optimized according to the high porosity characteristic of kiwifruit vine canopies. Scientific pruning should be adopted to regulate canopy ventilation and light transmittance, improving nitrogen uptake and utilization efficiency and further enhancing the precision and effectiveness of nitrogen nutrition regulation. This nitrogen inversion heatmap enables refined visualization of the spatial distribution of canopy nitrogen in mountainous kiwifruit orchards, driving the transition from traditional “uniform whole-area” field management to mountain-adapted “precision-targeted” nitrogen nutrition regulation. It provides intuitive and reliable technical support for precision fertilization and refined field management in the study area, as well as for other low-latitude and high-altitude mountainous kiwifruit orchards.

## 4 Discussion

This study focused on canopy nitrogen inversion of kiwifruit in the mountainous regions of Zhaotong, Yunnan Province. By integrating UAV multispectral technology with ensemble learning algorithms, we systematically investigated spectral variable screening, model construction, and optimization pathways. The objectives were to clarify the mechanisms underlying core features and the adaptation laws of inversion models, thereby providing technical solutions and theoretical support for precision nutrient management in mountainous orchards.

### 4.1 Correlation of spectral variables with nitrogen content

The sensitivity of spectral variables is a core prerequisite for determining the accuracy of nitrogen inversion. In this study, Pearson correlation analysis combined with two-tailed significance tests was used to systematically quantify the linear correlation strength and statistical validity between 25 spectral variables and the canopy nitrogen content of kiwifruit. The results showed that 2 single-band reflectance values and 9 vegetation indices were significantly correlated with nitrogen content. Among them, the GREEN band exhibited an extremely significant correlation with nitrogen content, and indices such as DVI, NDVI, and CARI also showed extremely strong and significant correlations. These variables collectively formed a core feature set adapted to the canopy structural characteristics and mountainous habitat of kiwifruit, providing a reliable input basis for the subsequent construction of nitrogen inversion models.

From the fundamental principles of crop nutritional physiology and spectroscopy, the high sensitivity of the GREEN band to nitrogen originates not only from the direct regulatory effect of nitrogen on the spectral characteristics of the plant canopy but also from the band’s natural adaptability to the heterogeneous light environment in mountainous areas. As a core nutrient for plant growth and development, nitrogen content directly affects the cellular structure and physiological activity of canopy leaves, thereby altering the absorption and reflection patterns of the canopy across different light bands. The study area is a low-latitude, high-altitude mountainous region where undulating terrain creates an interspersed distribution of sunspots and shadows. Compared with the red and red-edge bands, the GREEN band demonstrates stronger robustness to light interference, and its spectral signal is less likely to be masked by noise from the mountainous environment. Under sufficient nitrogen supply, the plant canopy grows vigorously with dense leaf tissue and significantly enhanced chlorophyll synthesis, strengthening the absorption of green light and correspondingly reducing canopy green-light reflectance. Conversely, under nitrogen deficiency, canopy growth is inhibited, leaf physiological activity declines, chlorophyll content decreases, and green-light reflectance increases significantly. This dynamic response between nitrogen content and green-light reflectance, combined with the adaptability of the GREEN band to mountainous environments, enables it to accurately characterize the dynamic changes in canopy nitrogen nutrition of mountain-grown kiwifruit (Zhuang et al., 2025).

As the primary driving feature for model prediction, DVI shows a significantly positive correlation between its standardized value and SHAP value. As a linear difference index of the near-infrared and red bands, DVI avoids signal distortion of ratio indices in mixed pixels of kiwifruit’s high-porosity canopy, has good tolerance to mild soil-canopy spectral mixing in mountain orchards, and can directly and accurately quantify plant nitrogen accumulation. NDVI enhances nitrogen-related spectral signals through the combined calculation of near-infrared and red bands, weakens the spatial heterogeneity of light intensity caused by mountainous terrain undulations via band ratioing, and reduces noise interference from bare soil background and topographic shadows in mountain orchards. DVI, NDVI, CARI, and other features are functionally complementary. In particular, CARI integrates difference information from the red-edge, green, and red bands; the red-edge band is more sensitive to subtle changes in leaf nitrogen in kiwifruit. Multi-band combined calculation effectively removes the dual interference of mountain shadows and soil background, significantly improving the feature set’s ability to capture nitrogen information and resist interference from complex mountain environments. These results fully demonstrate the scientific rationality and superiority of the multi-feature collaborative screening used in this study and verify the enhancement effect of band-combination indices on nitrogen-related spectral signals.

Traditional soil-adjusted vegetation indices such as OSAVI and SAVI did not reach statistically significant levels of correlation with kiwifruit canopy nitrogen content. This phenomenon resulted from the combined effects of the vine growth characteristics of kiwifruit and the complex mountain environment, rather than a single factor. As a typical vine fruit tree, kiwifruit has a highly porous canopy structure formed by interlaced branches and leaves, which already introduces substantial soil-canopy spectral mixing. The mountainous terrain undulations in the study area further intensify the spatial heterogeneity of mixed pixels, pushing the degree of soil-canopy spectral mixing well beyond the correction threshold of traditional vegetation indices. The fixed soil correction coefficients of these indices were designed for continuous canopies of field crops in plain areas and cannot dynamically match the highly heterogeneous soil interference in mountain kiwifruit orchards; instead, they dilute nitrogen-related spectral signals through over-correction. Meanwhile, their radiative transfer logic is based on continuous canopies of herbs or arbors and cannot adapt to the spectral reflection patterns of kiwifruit’s high-porosity canopy. Consequently, such indices struggle to effectively remove soil noise and extract pure canopy nitrogen information. These results inversely confirm the rationality of the targeted selection of features such as DVI and CARI in this study. These indices require no fixed coefficient correction or achieve interference removal through multi-band combination, better resist disturbances such as spectral variation and canopy porosity heterogeneity caused by mountainous terrain, and are more suitable for nitrogen inversion research on vine fruit trees in mountainous regions.

Existing studies on nitrogen inversion of plain kiwifruit mostly regard NDVI as the core sensitive index but neglect the adaptive value of the GREEN band to the heterogeneous light environment in mountainous areas (Kokkora et al., 2023), while soil-adjusted indices such as OSAVI and SAVI, though stable in nitrogen inversion of field crops in plain areas (Stefaniak and Łata, 2023), lose their effectiveness in mountainous vine fruit trees due to the inability to match canopy and topographic characteristics, which also confirms the necessity of screening exclusive spectral features for mountainous scenarios in this study.

### 4.2 Comparison and analysis of different modeling algorithms

Single-model inversion results indicate that PLSR achieves the best overall performance, which is significantly superior to LR and SVR. The core advantage of PLSR lies in its ability to effectively mitigate multicollinearity interference in high-dimensional spectral data through feature dimensionality reduction. It exhibits high adaptability with no obvious overfitting, as verified by 5-fold cross-validation, which is consistent with the stable performance of PLSR in nutritional parameter inversion studies of similar crops. For the BSR model, 12 core features are retained through endogenous feature screening, achieving a balance between fitting accuracy and generalization stability. In contrast, SVR exhibits significant overfitting due to its high sensitivity to hyperparameters and susceptibility to high-dimensional spectral noise—a common phenomenon in high-dimensional spectral data modeling.

The ensemble learning strategy significantly improves nitrogen inversion performance, with all three ensemble models outperforming the optimal single model (PLSR). Among them, the Boosting ensemble model achieves the best overall performance, with a 20.6% reduction in RMSE and an 11.1% improvement in RPD compared to PLSR. Based on a serial iterative weighting mechanism, the model assigns higher weights to mispredicted samples from the previous round, which enhances the ability to capture core feature information. This makes it particularly suitable for scenarios with high spectral signal variability and canopy structure heterogeneity in mountainous kiwifruit orchards. SHAP interpretability analysis confirms that the Boosting model can accurately identify the contribution of core features, and its prediction logic is highly consistent with spectroscopic principles and crop physiological mechanisms. The Stacking model integrates the advantages of linear and nonlinear models through hierarchical modeling, exhibiting robust performance but slightly inferior to Boosting. The Bagging model is not optimized for outliers, and mean fusion of multiple base learners fails to completely avoid noise interference in the training set, resulting in slightly weaker generalization stability than Boosting. This result indicates that the Boosting strategy, which focuses on error optimization, is more suitable for complex mountainous scenarios than the parallel resampling-based Bagging strategy. The core reason is that Boosting has stronger resistance to spectral variation and canopy heterogeneity induced by terrain. Existing studies on kiwifruit nitrogen inversion mostly use single models such as PLSR and RFR for inversion in plain scenarios (Kokkora et al., 2023), and ensemble learning algorithms have not been applied in nitrogen inversion of mountainous kiwifruit, while the inversion accuracy of the Boosting model constructed in this study is significantly higher than that of single models for plain kiwifruit and ensemble models for mountainous herbaceous/arbor crops (Pandey et al., 2022; Zhuang et al., 2025).

### 4.3 Research implications and applications

Spectral signal variation and canopy structure heterogeneity induced by topographic relief in mountainous kiwifruit orchards have long been a research gap in accurate nitrogen inversion. Most existing studies focus on field crops or plain orchards, and no adaptive technical solutions have been developed for low-latitude, high-elevation mountainous scenarios. In this study, a technical framework combining multi-feature screening and Boosting ensemble learning was constructed to break through the inversion bottleneck in mountainous environments. UAV flight technology effectively ensures the spatial consistency of spectral data in complex terrain, while the serial iterative weighting mechanism of the Boosting algorithm enhances the ability to capture core feature information. These two components synergistically overcome interference caused by topographic undulation and canopy porosity heterogeneity, significantly improving nitrogen inversion accuracy and further optimizing the nitrogen inversion technical system for mountainous vine fruit trees. This provides a new technical reference for nutrient monitoring in similar mountainous orchards.

The nitrogen heatmap generated based on the Boosting model achieves precise visualization of nitrogen spatial heterogeneity, clearly delineating nitrogen-rich, medium, and deficient zones, and providing intuitive and reliable technical support for orchard precision targeted fertilization. This technical outcome can guide growers to implement zonal control according to nitrogen levels in different regions: targeted nitrogen fertilizer supplementation in nitrogen-deficient areas; appropriate reduction of nitrogen fertilizer application in nitrogen-rich areas to avoid uncontrolled vegetative growth and agricultural non-point source pollution; optimization of irrigation regimes and planting density in medium-nitrogen areas to improve nitrogen use efficiency. This regulatory model not only reduces fertilizer waste caused by excessive fertilization, but also mitigates agricultural non-point source pollution, which is consistent with the core development concept of smart agriculture—cost reduction, efficiency enhancement, greenness, and sustainability. In addition, the precise identification of micro-scale nitrogen differences by the heatmap provides key technical support for the implementation of small-plot zonal management and precision management models in mountainous orchards.

### 4.4 Future prospects

Although this study provides an effective technical solution for accurate canopy nitrogen inversion in mountainous kiwifruit, several limitations require further improvement. (1) Sample coverage is relatively limited: the study only focuses on the fruit expansion stage of kiwifruit, does not include other mainstream varieties, and does not involve spectral response differences during key reproductive stages (e.g., budding and flowering). This results in the need for further verification of the model’s variety adaptability and temporal stability. (2) Spectral data dimensionality is limited: the study relies solely on four discrete bands (green, red, red edge, and near-infrared) from a single multispectral sensor, lacking fine spectral information from continuous hyperspectral bands. This makes it difficult to accurately capture the weak response correlation between nitrogen content and spectral signals. (3) Consideration of influencing factors is insufficient: the interference effects of soil physicochemical properties, regional precipitation distribution, and field management practices on nitrogen inversion results were not quantified, which may affect the model’s robustness under complex mountainous conditions.

In response to the above limitations, future research can be advanced in three directions. (1) Expand sample coverage to include spectral and nitrogen content data from different representative varieties and the entire growth cycle, and construct an integrated variety-specific and temporally adaptive inversion model to improve the model’s universality and temporal stability. (2) Integrate multi-source data, combining ground-based hyperspectral data, soil physicochemical parameters, and Internet of Things (IoT) real-time monitoring data to build a high-precision multi-source fusion inversion model, thereby further improving inversion accuracy and anti-interference capability under complex mountainous conditions. (3) Optimize the model architecture by introducing deep learning algorithms coupled with an attention mechanism to enhance the accurate extraction of core features (e.g., DVI and CARI). Simultaneously, integrate transfer learning technology to realize rapid model adaptation across different mountainous orchard scenarios, providing more efficient technical support for precision nutrient management in mountainous orchards.

## 5 Conclusions

This study focuses on the characteristics of mountainous scenarios and conducts accurate canopy nitrogen inversion research on mountainous kiwifruit in Zhaotong, Yunnan Province, by integrating UAV multispectral technology and ensemble learning algorithms. The core conclusions are as follows:

1. Eleven nitrogen-sensitive spectral variables were screened, among which DVI, the GREEN band, NDVI, NDWI, and CARI exhibit a highly significant correlation with nitrogen content. Validated by SHAP analysis, these variables collectively form an efficient characterization system tailored to the canopy characteristics of kiwifruit in mountainous areas.
2. Among the five single models, PLSR achieves the best overall performance. It mitigates multicollinearity through feature dimensionality reduction, strikes a balance between fitting accuracy and generalization stability, and is well-suited for high-dimensional spectral data inversion.
3. The Boosting ensemble model delivers the optimal inversion performance, with significantly improved performance compared to PLSR. Its serial iterative weighting mechanism enhances core feature capture, enabling effective adaptation to spectral variation and canopy heterogeneity in mountainous areas, and exhibits excellent stability and anti-interference capability.
4. The nitrogen heatmap generated based on the Boosting model clearly depicts the spatial distribution pattern of nitrogen, providing a scientific basis for precision zonal fertilization and aligning with the development orientation of green agriculture.

This study verifies the feasibility of coupling UAV multispectral technology with Boosting ensemble learning for nitrogen inversion in mountainous kiwifruit, further optimizing the precise nitrogen inversion technical system for mountainous vine fruit trees. The complete technical pathway constructed in this study—encompassing feature screening, model construction, integration optimization, and visualization application—provides solid theoretical support and practical technical reference for the large-scale promotion of smart agriculture in mountainous orchards.

## Data Availability

The raw data supporting the conclusions of this article will be made available by the authors, without undue reservation.
